# Stunting and associated factors among 6–23 month old children in drought vulnerable kebeles of Demba Gofa district, southern Ethiopia

**DOI:** 10.1186/s40795-022-00501-2

**Published:** 2022-01-26

**Authors:** Tuba Tringo Tadele, Chameno Chalite Gebremedhin, Makiso Urugo Markos, Endale Liben Fitsum

**Affiliations:** 1 College of Engineering and Agro-Processing Technology, Arbaminch University, Arba minch, Ethiopia; 2 College of Agricultural Sciences, Arbaminch University, Arba minch, Ethiopia; 3 College of Agricultural Sciences, Wachemo University, P.O.BOX: 667, Hosaena, Ethiopia; 4 College of Agriculture and Veterinary Medicine, Jimma University, P.O.BOX: 307, Jimma, Ethiopia; 5 College of Medicine and Health Sciences, Wachemo University, Hosaena, Ethiopia

**Keywords:** Dietary diversity, Food insecurity, Children and Stunting

## Abstract

**Background:**

Stunting is impaired linear growth of children: they experience stunting in the first 1000 days after conception and is an indication of chronic malnutrition. Children under the age of two are regarded as the most vulnerable to malnutrition due to their rapid growth and greater exposure to infectious disease.

**Objective:**

To assess the magnitude and associated factors of stunting among 6 to 23-month-old children in drought-vulnerable kebeles of the Demba Gofa district, southern Ethiopia.

**Methods:**

A community-based cross-sectional study was conducted from February to March 2021. Systematic random sampling was used to select pairs of mothers/caregivers with children aged 6 to 23 months. A semistructured questionnaire and anthropometric measurement were used to collect the data. The data were checked coded and entered into Epi-data version 3.1 and exported to SPSS for Windows version 20.0 for analysis. Simple and multivariable linear regressions were conducted. The level of significance was declared at 95% CI and *p-value* < 0.05.

**Results:**

The magnitude of stunting in the study area was 79(21.82%). Household dietary diversity [β = 0.217, 95% CI, 0.093–0.342], early initiation of complementary feeding [β = 0.444, 95% CI, 0.344–0.543], frequency of breastfeeding within 24 h [β = 0.217, 95% CI, 0.179–0.263] and child eating animal source food [β = 0.351, 95% CI, 0.196–0.506] were positively significant predictors of child height/length-for-age (HAZ).

**Conclusion:**

The extent of stunting in the study area is relatively lower than that in regional and national reports, but one out of five children were still stunted. Therefore, health education on infant and young child feeding practices should be provided to mothers to reduce the problem.

**Supplementary Information:**

The online version contains supplementary material available at 10.1186/s40795-022-00501-2.

## Introduction

A child with height/length-for-age (H/LAZ) below -2 standard errors compared with the reference group (Z-score < -2) is considered to be stunted [1]. Stunting is impaired linear growth of children that they experience it in the first 1000 days after conception. It is an indication of chronic malnutrition in early childhood [2]. Children under the age of two are regarded as the most vulnerable to malnutrition due to their rapid growth and greater exposure to infectious disease. Approximately 70% of stunting occurs in this period [3].

Globally, 21.9% (149 million) of under-fives are stunted, and of these, 33.6% account for East Africa [4]. Similarly, the prevalence of stunting in Africa among 6 to 23-month-old children ranges from 61.5% in the Central Africa Republic [5] 51% in Kenya [6] and 20.3% in Egypt [7]. In Ethiopia, stunting is still a major public health concern, and 37% of children below five years are stunted. Regionally, the magnitude of stunting in Southern Nations and Nationalities is close to the national average, and recent data indicate that 36% of children below 5 years of age in the region are stunted [8]. Similarly, studies conducted in different parts of Ethiopia show that the prevalence of stunting among children aged 6–23 months is 58.1% in Dabat [9], 18.7% in Kemba District [10] and 36.2% and 42.6% in two agroecological areas in the northern and eastern parts of Ethiopia, respectively [11].

Approximately 10.5 million children die each year as a result of malnutrition, with 98% of these deaths occurring in developing countries [12]. In children and adolescents, stunting is linked to poor physical and cognitive development, as well as poor academic achievement [13, 14]. Stunted individuals will earn an average of 22% less than their non-stunted counterparts [15]. Study findings show that young children who were stunted are 33% less likely to escape poverty as adults, resulting in overall gross domestic product (GDP) loss of 4 to 11 percent in Africa and Asia [16, 17]. In Ethiopia, the cost of hunger as a result of malnutrition is estimated to be up to 16.5% of the GDP annually [18].

Stunting is mainly associated with poor maternal nutritional status and other interrelated problems, such as dietary intake, infectious diseases, micronutrient deficiencies and the environment [2, 3, 19, 20]. Furthermore, socioeconomic, demographic and environmental factors concomitantly have a significant effect on stunting [21]. In addition, food insecurity reduces food consumption dietary energy intake and compromises diet quality and diversity. Deprivation of calories or essential nutrients can erode both physical and mental health, which leads to less economically productive populations [22, 23]. The severe food insecurity of households in Bangladesh and Ethiopia has increased the risk of being stunted [23].

Food insecurity, poor complementary feeding, lack of potable water, poor housing conditions and high burden of illness that accompany chronic poverty create an environment that can negatively affect children’s educational attainment and economic security in the long term in dwellers of low/middle income countries [15, 24]. Communities in food insecure areas are susceptible to insufficient access to nutritionally adequate, safe food and inadequate utilization of food. Consequently, it reduces dietary variety and nutrient intake and affects the nutritional status of inhabitants. In addition, an unsanitary environment that exposes children to repeated infections leads to poor absorption or utilization of the nutrients consumed [23]. Some kebeles of Demba Gofa district are identified as drought vulnerable, food insecure and incorporated in safety net programs by legislative body of the district and obtain humanitarian emergency supports.

Additionally, environmental sanitation is deprived, and clean drinking water problems are evident in food-insecure areas of the district: these problems are likely to increase the frequency of stunting in the area. Stunting often goes unrecognized in communities where short stature is so common that it is considered normal. The difficulty in visually identifying stunted children and the lack of routine assessment of linear growth in primary health care services explain why it has taken so long to recognize the magnitude of this hidden scourge [25]. Additionally, once established, stunting is irreversible, which is why this study specifically focuses on stunting. Furthermore, there is limited evidence on the magnitude of stunting among 6 to 23-month-old children in the proposed study area. In addition, in identifying determinants of stunting in the drought-vulnerable areas of the district, there is also a paucity of evidence regarding determinants of stunting. Therefore, this study aims to identify the extent and determinants of stunting among children between 6 to 23 months of age in drought-vulnerable kebeles of the Demba Gofa district.

This study helps to identify the hidden level of stunting in drought-vulnerable areas. It also helps policy makers and program planners emphasize and act to minimize malnutrition prevalence, particularly in drought-vulnerable areas, and the burden at the national level.

## Methods

### Study area and period

**Fig. 1:**
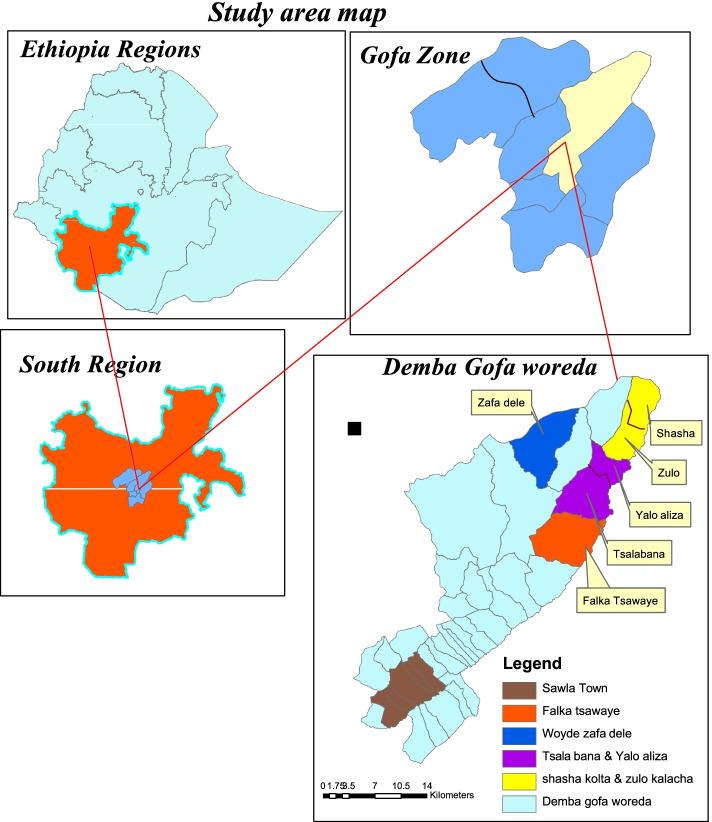
Map of the study area

Demba Gofa District is one of eight districts located in the Gofa Zone in Southern Nations Nationalities and the Regional State of Ethiopia. The district location lies between 8°71′81″ north and 43°89′85″ east. The district is located 305 km away from the regional capital city Hawassa and 525 km southwest of Addis Ababa (Fig. 1). The study was conducted from February to March 2021.

### Study design and population

A community-based cross-sectional study was conducted among residents in Demba Gofa district, Ethiopia. The study population was mothers/caregivers paired with children 6 to 23 months old who lived in the selected kebeles for at least 6 months. Those who had mental illnesses interfering with the interview were not considered in the study. Children above 23 months and below 6 months were also excluded from this study.

### Sample size and sampling technique

Sample size was determined using Epi-Info software version 7.2.2.6 with the following assumptions. The total number of children aged 6 to 23 months in the selected kebeles was 713, the proportion of regional stunting was 36% [8], the confidence interval (CI) was 95%, and the acceptable margin of error was 5%. After adding 5% for nonresponse, the final sample size becomes 362.

**Fig. 2:**
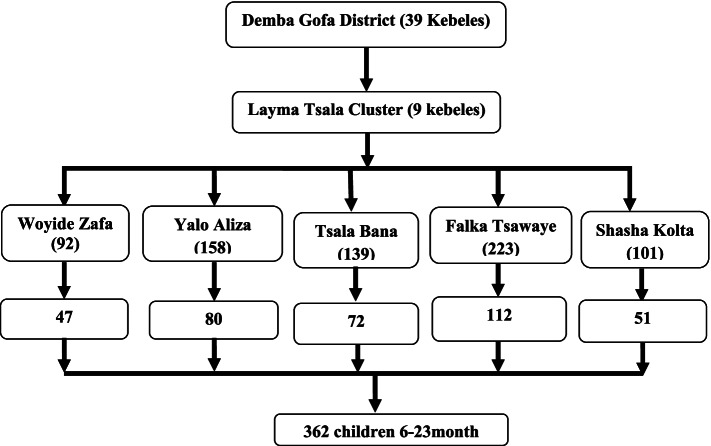
Schematic representation of the sampling technique to select the study participants, 2021

Primarily, the total number of children in the drought-vulnerable kebeles (small administrative unit) of Demba Gofa district was identified from each kebele’s health extension logbook. Then, the residential house of children 6 to 23 months old age was identified and coded. Then, systematic random sampling with proportional allocation was used to select infant mother/caregiver pairs from each kebele. Specifically, study participants were selected systematically according to their arrangement of the households every (k = 2) interval. Finally, if there was more than one child below the age of five within the household, one child was selected randomly (Fig. 2).

### Data collection and measurements

The data were collected from both primary and secondary sources of data. Primary data were collected using a structured and pretested interviewer-administered questionnaire developed to collect information on sociodemographic, maternal and child health, environmental characteristics, household dietary diversity and anthropometric measurements. Secondary data related to child-care practices (breastfeeding, complementary feeding practices), maternal characteristics (number of children, feeding practice during pregnancy and lactation), and facilities (drinking water) were collected using a pretested semistructured questionnaire from the Demba Gofa district agricultural and natural resource department and from the district health office. One health extension worker and one nurse per kebele were allocated to collect the data.

Household dietary diversity (HDD) data were collected according to Food and Agricultural Organization (FAO) guidelines [26] by using semistructured questionnaires and microlevel data drawn from repeated 24-h diet recall surveys for daily dietary intake of household members. An individual who was responsible for preparing food or serving food for the family members was used as a source of household dietary diversity data. Household dietary diversity was assessed with a scale of seven food groups: cereals and grains, vegetables, fruits, dairy products, oil and fat, and protein-rich and discretionary calorie foods. Finally, the HDD score was found to be optimal when a child was fed foods greater than four food groups per day [27].

For animal food source (ASF) consumption, data analysis was conducted according to the method employed by Krasevec et al. [28]. The food lists on household dietary diversity questionnaire grouped into three categories of animal source foods as: (1) milk products including infant formula, yogurt cheese, butter and other locally available milk products: (2) meat group that includes any animal organ meat, poultry and fish: (3) eggs. Finally, animal source food consumption data were constructed as categorical based on the number of animal source food consumed, with a minimum zero and maximum of three [28].

Anthropometric data were collected according to the method designed by Gibson [29]. The child health card or birth certificate was used to ascertain and record the age of the index child. In situations where the mother/caregiver did not have the documents to ascertain the age of the child, they were asked to identify a child from the neighbourhood who was born almost the same time. The length was measured in the recumbent position using a sliding board by two data collectors and taken to the nearest 1 mm. Length was measured based on the standard by keeping the child sight perpendicular to the roof and the knees well stretched [29]. Measurement was taken twice, and the average result was taken to ensure accuracy.

The height-for-age index of children was calculated using growth standards published by the World Health Organization (WHO) in 2006. These growth standards were generated through data collected in the WHO Multicentre Growth Reference Study [30] and expressed in standard deviation units from the Multicentre Growth Reference Study median. The height-for-age index is an indicator of linear growth retardation and cumulative growth deficits in children. Children with height-for-age Z-scores below minus two standard deviations (− 2 SD) from the median of the WHO reference population were considered to be stunted or chronically malnourished, while children who were below minus three standard deviations (− 3 SD) from the reference median were considered severely stunted. However, the dependent variable HAZ was considered a continuous variable in the analysis.

### Data quality management

The questionnaire was first translated into the local language Amharic for common understanding and retranslated back to English to check its consistency. A pretest was conducted on 5% of the households before the actual data commencement. Question sequence arrangement was performed based on the pretest results. One day of training was given for data collectors and supervisors on the objective of the study and the measurement.

### Data analysis

Data were checked, coded and entered into Epi-data version 3.1 and exported to SPSS version software v.20.0 for analysis. First, frequencies and proportions were computed to present descriptive results. Anthropometric data analysis WHO Anthro v.3.2.2 software was utilized to convert raw nutritional data into Z-scores and then transferred to SPSS version 20. Normality, equal variance (homoscedasticity), linearity assumptions and multicollinearity were checked before fitting the linear regression model.

Accordingly, the assumption of linearity was checked through both scatter plots and correlation matrices and was satisfied. The assumption of normality was checked by plotting P-P plots and Kolmogorov–Smirnov and Shapiro–Wilk tests, and it was also satisfied. The assumption of homoscedasticity was satisfied by plotting a scatter plot of standardized residuals against the standardized predicted values, and it was randomly distributed. Durbin Watson statistics were used to check the assumption of independence of errors and autocorrelations. The value of the Durbin Watson statistics for these data was 1.87, which falls within the acceptable range from 1.50 to 2.50: therefore, this analysis satisfied the assumption of independence and no autocorrelations. Multicollinearity was also checked using whether the standard error was < 2, variance inflation factor (VIF) < 10, tolerance > 0.1. Hence there was no evidence of Multicollinearity.

Simple linear regression analysis was carried out to identify the variables associated with the outcome variable. Variables with *p-values* less than 0.2 in the simple linear regression analyses were considered candidate variables for multivariable linear regression. To declare statistical significance on multivariable linear regression, the regression coefficient at 95% CI with a *p*-*value* less than 0.05 was used. The R-square was used to report the model fitness.

## Results

### Socio-demographic characteristics

The sociodemographic characteristics of the study population indicated in Table 1 show that all of the study participants were Gofa by their ethnicity. Regarding their religion, 267(73.7%) were Protestant, and the remaining 95(26.3%) were Orthodox. One-third of 122(33.7%) mothers participating in this study were 18–45 years old, almost all of whom 360(99.4%) were married, and 355(98%) of the households were headed by the husband. In terms of family size, 216(59.5%) study participants had 3–5 family members. Half of the mothers, 182(50.3%) and 204(56.4%) husbands, participated in the study and had followed formal education.

**Table 1:** Sociodemographic characteristics of the respondents in Demba Gofa district, Ethiopia, 2021

Variables	Response variables	Frequency	Percent
**Ethnicity**	Gofa	362	100
**Religion**	Protestant	267	73.7
	Orthodox	95	26.3
**Mothers age**	18–25	122	33.7
	26–40	236	65.2
	> 41	4	1.1
**Marital status**	Married	360	99.4
	Single	1	0.3
	Widowed	1	0.3
**Household head**	Husband	355	98
	Wife	7	2
**Family size**	3–5	216	59.5
	6–9	137	38
	10–12	9	2.5
**Children below 5 years of age per household**	1	144	39.8
	2	187	51.6
	3	31	8.6
**Mother’s followed formal education**	Yes	182	50.3
	No	180	49.7
**Husband’s followed formal education**	Yes	204	56.4
	No	158	43.6
**Mother’s occupation**	Housewife	356	98.3
	Employee	2	0.6
	Merchant	4	1.1
**Husband’s occupation**	Farmer	319	88.1
	Merchant	30	8.3
	Employee	13	3.6
**Monthly income of the household**	100–500	338	93.4
	501–1000	12	3.3
	> 1000	12	3.3
**Households with livestock**	Yes	267	73.8
	No	95	26.2
**Households with agricultural farm land**	Yes	189	52.2
	No	173	47.8

### Maternal and child health and environmental characteristics

Of the 362 children, 226(62.5%) were 12 to 23 months old. Out of them 194 (53.6%) were males. Regarding antenatal care, half of the 196 mothers (54.1%) participated in this study and followed antenatal care during their pregnancy period. Seventy-five percent of the mothers delivered their children at home. Most of the children (291, 80.4%) did not exclusively breastfeed for 6 months. Only 14(3.9%) of the mothers initiated complementary feeding in a timely manner, and 23.8% began complementary feeding at the 3^rd^month of age. Less than half of the children 161(44.5%) fed fruit and vegetable source foods, and 139(38.4%) fed animal source foods. Sixty percent of the households had more than one child below the age of five years (Table 2).

**Table 2:** Maternal and child and environmental characteristics of the respondents in Demba Gofa district, Ethiopia, 2021

Variables	Response variables	Frequency	Percent
**Age (months)**	6–11	136	37.5
	12–23	226	62.5
**Gender of the child**	Male	194	53.6
	Female	168	46.4
**Antenatal care utilization**	Yes	196	54.1
	No	166	45.9
**Extra meal during pregnancy**	Yes	173	47.8
	No	189	52.2
**Extra meal during breastfeeding**	Yes	258	71.3
	No	104	28.7
**Place of delivery**	Health institution	90	24.9
	Home	272	75.1
**Squeezed out first milk**	Yes	114	31.5
	No	248	68.5
**Breast milk initiation**	In the first one hour after delivery	295	81.5
	After an hour	56	15.5
	After days	11	3.0
**Daily breast feeding rounds (frequency of breastfeeding in 24 h)**	5–8	273	75.3
	9–12	86	23.7
	> 12	3	0.9
**Exclusive breast feeding**	Exclusively fed	71	19.6
	Not fed exclusively	291	80.4
**Initiation of complementary feeding**	At 3^rd^ Month	86	23.8
	At 4^th^ Month	167	46.1
	At 5^th^ Month	95	26.2
	At 6^th^ Month and Above	15	4.2
**Feeding the child fruit and vegetables**	Yes	161	44.5
	No	201	55.5
**Feeding the child animal source food**	Yes	139	38.4
	No	223	61.6
**Child received deworming drug**	Yes	32	8.8
	No	330	91.2
**Source of drinking water**	Protected spring water	12	3.3
	Unprotected spring water	55	15.2
	Public stand pipe (tap) water	272	75.1
	Runny water	23	6.4
**Availability of hand washing facility**	Fully functional	61	16.9
	Partially functional	188	51.9
	Not available	113	31.2
**Children have diarrhoea in the last two weeks**	Yes	122	33.7
	No	240	66.3
**Vaccination status of the children**	Completed	132	36.5
	Not completed	116	32.0
	Not vaccinated at all	9	2.5
	Being vaccinating	105	29.0

### Household dietary diversity score of respondents

**Fig. 3:**
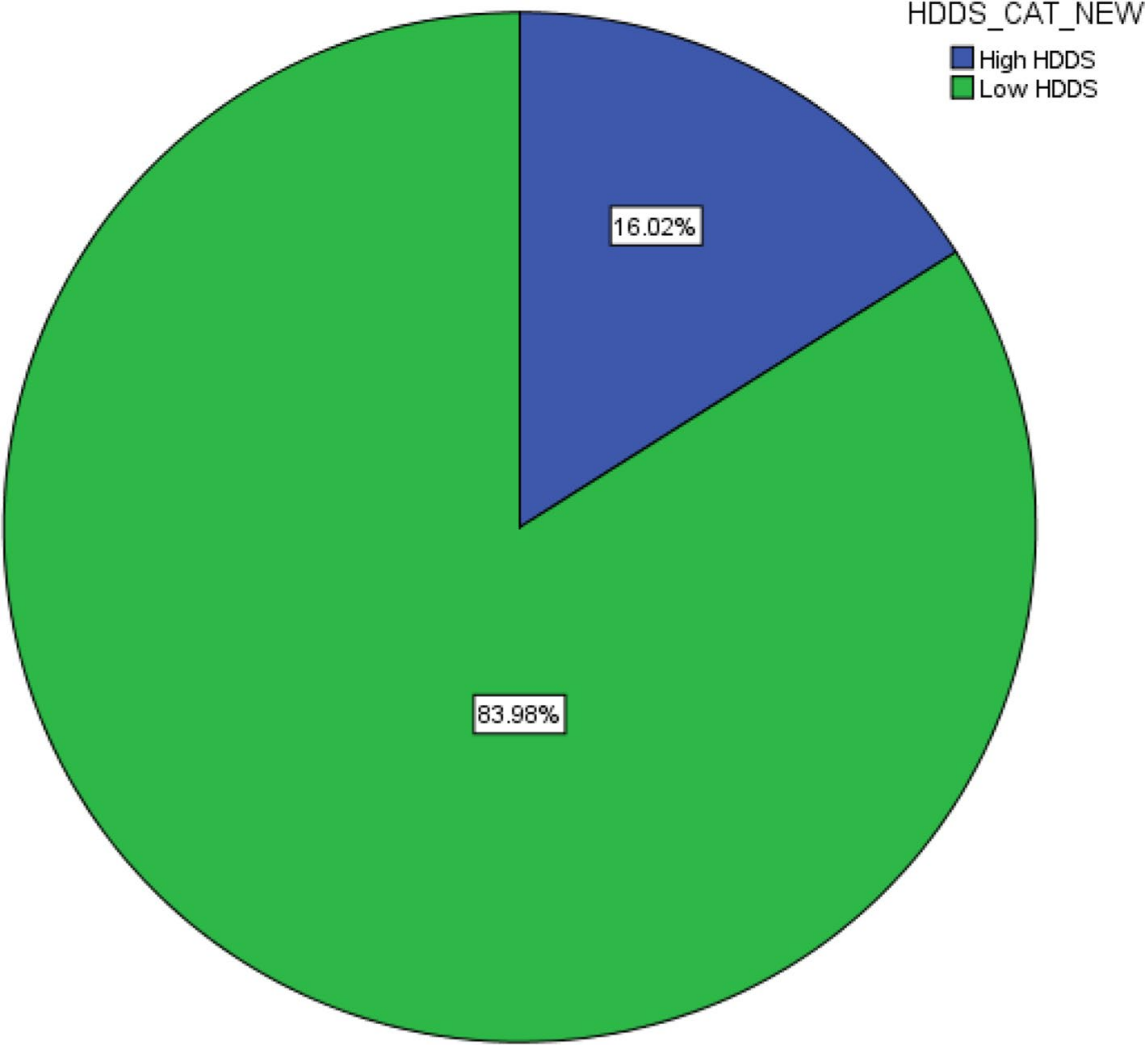
Household dietary diversity score of the respondents in Demba Gofa district, Ethiopia, 2021

According to the study results, 304(84%) households had low dietary diversity scores and ate fewer than 4 food groups. However, 58(16%) households scored high household dietary diversity scores and ate more than or equal to four food groups (Fig. 3).

### Prevalence of child stunting

**Fig. 4:**
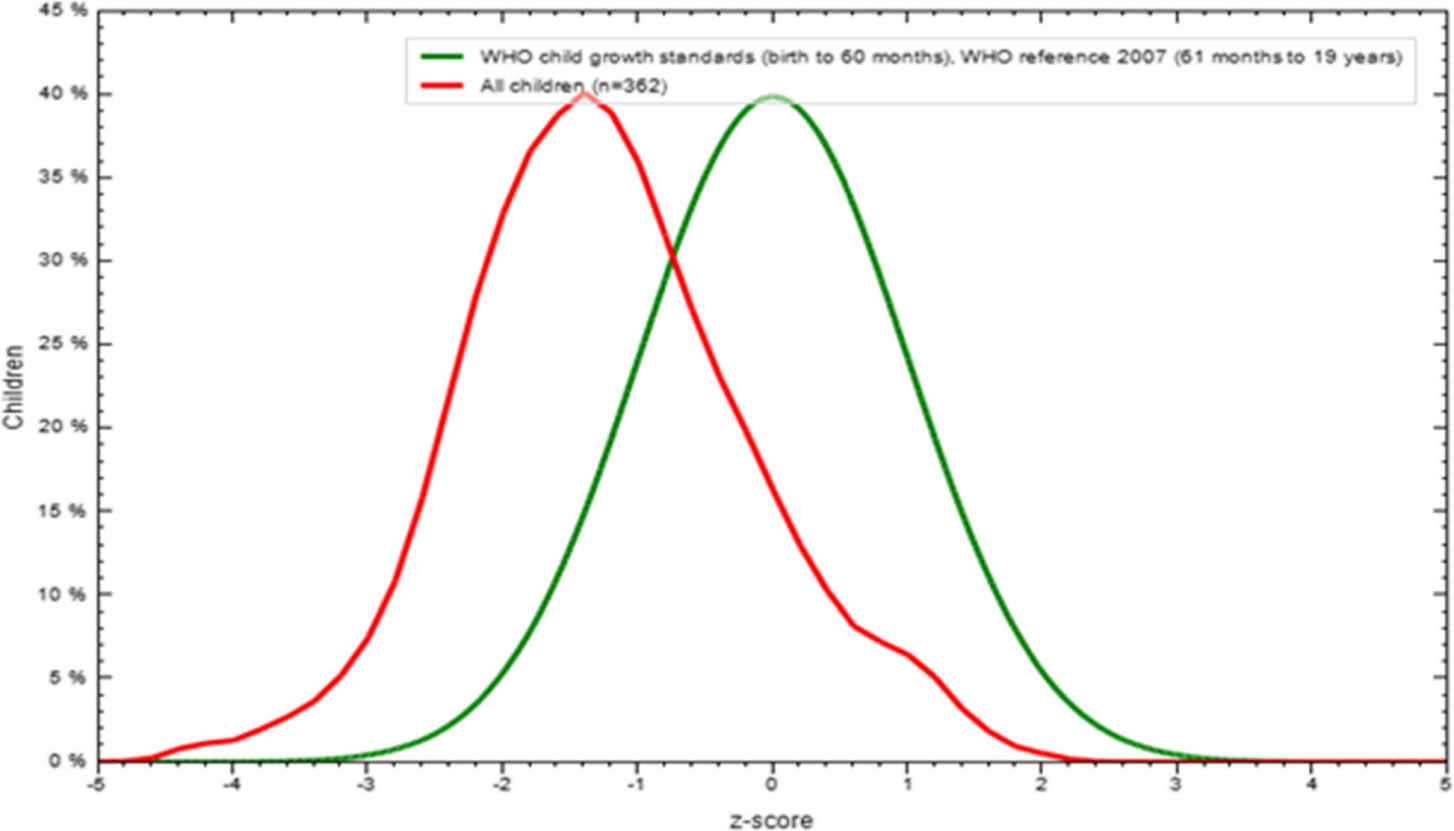
Height-to-age Z-score of study children in relation to WHO reference group

Among the approached children, the output indicated in Fig. 4 and Table 3 showed that 79(21.82%) children’s height-for-age was < -2 standard errors compared with the WHO reference group (Z-score < -2). The magnitude of stunting was more prominent among males (49, 62%) than females.

**Table 3:** Height-to-age children 6–23 months old in Demba Gofa district, Ethiopia, 2021

**Height-for-age**	Male		Female		Both Sexes		Total
	6–11 month *n* = 72	12–23 month *n* = 122	6–11 month *n* = 64	12–23 month *n* = 104	6–11 month *n* = 136	12–23 month *n* = 226	
** < -3 SD %**	6(8.3%)	4(3.3%)	2(3.1%)	2(1.9%)	8(5.9%)	6(2.7%)	79(21.82%)
** < -2 SD %**	15(20.8%)	34(27.9%)	10(15.6%)	21(20.2%)	25(18.4%)	55(24.3%)	
**Normal**	51(70.9)	84(68.8%)	52(81.3%)	81(77.9%)	103(75.7%)	165(73%)	283(78.18%)

### Factors associated with child stunting

The multivariable analysis model explained 61.8% of the variance in the HAZ score (adjusted R square = 0.618, *P-value* < 0.001). Multivariable linear regression analysis showed that household dietary diversity, months of complementary feeding started, frequency of breastfeeding within 24 h and child feeding animal source food were significantly associated with child stunting.

Keeping the other variables constant, the dietary diversity score in the households increased by 0.217 with one unit, and the child HAZ increased by 0.217 [β = 0.217, 95% CI, 0.093–0.342]. As the child complementary feeding started period increases before six months with one unit HAZ of the child, it also increases by 0.444 [β = 0.444, 95% CI, 0.344–0.543]. When the frequency of breastfeeding within 24 h increases by one unit, the child HAZ increases by 0.221 [β = 0.217, 95% CI, 0.179–0.263]. As the children ate animal source food, the child HAZ increased by 0.351 compared to children who did not ate animal source food [β = 0.351, 95% CI, 0.196–0.506] (Table 4).

**Table 4:** Multivariable linear regression model predicting child HAZ in Demba Gofa district, Ethiopia, 2021

Variables	Unstandardized Coefficients		P-value	95% Confidence Interval for β		Collinearity Statistics	
	**β**	**Standard Error**		**Lower Bound**	**Upper Bound**	**Tolerance**	**VIF**
**Household dietary diversity**	.217	.063	.001^a^	.093	.342	.993	1.007
**Months of complementary feeding started before 6 month**	.444	.051	.000^a^	.344	.543	.667	1.500
**Frequency of breastfeeding within 24 h**	.221	.021	.000^a^	.179	.263	.646	1.548
**Child feeding animal source food**	.351	.079	.000^a^	.196	.506	.726	1.378

β** = **Regression coefficient

^a^Significant association

*VIF* Variance Inflation Factor

## Discussion

Adequate nutrition during infancy and early childhood is paramount for child growth, health and development. Stunting is a much more common nutritional problem that mainly affects developing countries such as Ethiopia. Even though the government of Ethiopia is striding to overcome child stunting with the target of “zero stunting” in 2030, it is still 37% in 2019 [8]. This indicates that the country is not on track to achieve the seated target if this reduction proportion per annum is continuous at this pace. Therefore, assessing the extent and predictors of linear growth in drought-vulnerable areas is essential.

The magnitude of stunting in the study area was 21.82%. The results of this study were similar to those of a study conducted in Kemba District in southern Ethiopia [10] and Egypt [7]. However, the finding is relatively lower than that of regional and national reports [8], studies conducted in the Central Africa Republic [5], Kenya [6], Dabat District [9], and a study conducted in two agroecological areas in the northern and eastern parts of Ethiopia [11]. In addition, the present study showed that stunting was more prevalent in male children (62%). A study finding by Forsido et al*.* [31] also found parallel results and identified that male children were 2.6 times [AOR = 2.601, 95% CI (1.681, 4.025)] more likely to become stunted than females [31]. Male children are more vulnerable to environmental stress than their counterparts [32, 33].

The household dietary diversity score of the study subjects was one of the variables that was significantly attributed to child height/length-for-age [β = 0.217, 95% CI, 0.093–0.342]. Of the total households, 84% scored low dietary diversity, and the majority (72.15%) of the total stunted children were from those households. This finding of our study was supported by a community-based cross-sectional survey in 42 countries among 6 to 23-month-old children that found a positive association (adjusted risk ratio = 1.15: 95% CI 1.01, 1.31) between dietary diversity and stunting [34]. Similarly, another cross-sectional survey conducted in Nigeria intended to assess the dietary diversity score and its association with nutritional status and socioeconomic characteristics of children below five years of age. The study identified that the dietary diversity score has a significant (*p* < 0.03) association with the height-to-age ratio of study participants [35].

Conversely, a study finding from Ghana on children 6–36 months of age comes up with different conclusions. The study assessed the link between dietary diversity and the nutritional status of children and depicted a lack of association between high, medium and low household dietary diversity score categories and stunting (AOR = 1.18, 95%, CI = 0.79, 1.76, *p* = 0.409) [36]. However, the present study area is primarily identified as drought vulnerable, food insecure and receiving humanitarian emergency support from the district, and being a food insecure area might be the main reason behind the low household dietary diversity score of the majority of households. Yang et al. [37] also reported the existence of a strong association between the food insecurity of households with lower dietary diversity scores and an increased prevalence of stunting. The food insecurity of households significantly (*p* < 0.001) lowered dietary diversity compared to food-secured households. Moreover, the food insecurity of households is associated with low dietary diversity and stunting [37]. Additionally, in our study area, the community depends on limited dietary sources, particularly maize, cassava and sorghum only.

In the present study, a significant positive association (*p* < 0.000) was observed between early introduction of complementary feeding before 6 months of age and child stunting [β = 0.444, 95% CI, 0.344–0.543]. The majority (93.37%) of the study participants started complementary feeding before six months of age, and 100% of stunted children were among those who started complementary feeding before 6 months of age. Only 6.63% of children initiated complementary feeding in a timely manner at 6 months, and none of them stunted. A recent cross-sectional study from Indonesia also found similar results to this finding and portrays that untimely introduction of complementary food was associated with stunting in children. Children who obtain their first food before six months of age are two times more likely to become stunted than their counterparts [38]. Likewise, a more recent study from Ethiopia also strengthened the idea that early initiation of complementary feeding before six months of age significantly (AOR = 1.58: 95%, CI, 1.07, 2.34) contributed to stunting in children [39].

In another way, this study revealed the frequency of breastfeeding within 24 h as a significant variable (*p* < 0.000) positively associated with height/length-for-age of the children. As the frequency of breastfeeding increases by one unit, the child linear growth increases by 0.221 [β = 0.217, 95% CI, 0.179–0.263]. A breastfeeding frequency > 12 times in 24 h is recommended as a reference, and children who breastfeed less than the recommended frequency are indicated as a risk for child stunting [40].

Eating animal source food during complementary feeding was positively (*p* < 0.000) associated with child stunting. Children who did not feed animal source food, such as milk and eggs, were 0.351 times [β = 0.351, 95% CI, 0.196–0.506] more prone to stunting than those who consumed animal source food. In the present study, 61.6% of the children did not receive animal source food during complementary feeding, and 96.2% of stunted children were among them. However, only 3.8% of stunted children are children who obtain ASF during their complementary feeding (38.4%). According to Headey et al. [41], intake of animal source food reduced stunting by 3.7–3.8 percentage points. The study was conducted on 130,432 children between 6–23 months old from 49 countries on animal source food consumption and its association with stunting. This finding shows that not consuming animal source food significantly increased the magnitude of stunting. Moreover, consuming animal source food significantly (*p* < 0.047) improved the height-for-age of 12 to 36-month-old children [41].

The study addressed an area that makes children vulnerable to malnutrition. The study used a 24-h recall to identify the dietary diversity score of the mothers. However, it does not include the acute form of malnutrition.

## Conclusion

This study identified the prevalence and predictors of stunting among 6 to 23-month-old children in drought-vulnerable kebeles of the Demba Gofa district in southern Ethiopia. The magnitude of stunting in the study area was relatively less than national and regional reports: however, still one out of five children were stunted. Household dietary diversity, early initiation of complementary feeding, frequency of breastfeeding within 24 h and child eating animal source food were found to be significantly associated with child height/length-for-age. Continuous health education on infant and young child feeding practices is necessary in the study area.

## Supplementary Information

**Additional file 1.**

**Additional file 2.**

**Additional file 3.**

## Data Availability

The datasets used or analysed during the current study are available from the corresponding author on reasonable request.

## Abbreviations

ASF: Animal Food Source
FAO: Food and Agricultural Organization
GDP: Gross Domestic Product
HAZ: Height-for-age
HDD: Household dietary diversity
VIF: Variance Inflation Factor
WHO: World Health Organization
