# Supplementary material for: Stunting and associated factors among 6–23 month old children in drought vulnerable kebeles of Demba Gofa district, southern Ethiopia
Source: BMC Nutr. 2022 Jan 26;8:9. doi: 10.1186/s40795-022-00501-2 (PMC8790906; doi:10.1186/s40795-022-00501-2)
Supplement: Supplementary file 1 — Additional file 1. [file 40795_2022_501_MOESM1_ESM.docx]

**Arbaminch University Sawla Campus**

**Department of Food Technology and Process Engineering**

**Anthropometric Data collection form 2013**

**Identification information**

Name of surveyor_____________________ Name of survey supervisor________________________

Name of village/community__________________ Cluster Number_________________________________

Interview date ____/______/________ Rescheduled interview date____/______/_________

Time of interview ___________________

Problems with interview (comment)__________________________________________________________

1. Name of Mother___________________________________________________________________
2. Name of child_____________________________________________________________________
3. Sex of Child Male Female
4. Date of measurement________________________________________________/_______/_______
5. Date of Birth of child________________________________________________/_______/_______
6. Age of mother (in month)_____________________________________________/_______/_______
7. Source used to verify child’s date of birth
8. Birth certificate
9. Health card
10. Estimated (Not verified with written document)
11. Other (specify)________________________________________________________
12. Stature
13. Recumbent Length (Children less than 24 months)____________________________cm
14. Clothes worn by the child or problems with measurements
15. No clothes and no problems
16. Light clothes
17. Heavy clothes or multiple layers
18. Braised in girl’s hair
19. Other (Specifiy)________________________________________________________________
20. Status of questionnaire
21. Questionnaire complete
22. Child refused to be measured
23. Mother refuse to have child measured
24. Other (specify)_________________________________________________________________

Comments_____________________________________________________________________
