# Supplementary material for: Stunting and associated factors among 6–23 month old children in drought vulnerable kebeles of Demba Gofa district, southern Ethiopia
Source: BMC Nutr. 2022 Jan 26;8:9. doi: 10.1186/s40795-022-00501-2 (PMC8790906; doi:10.1186/s40795-022-00501-2)
Supplement: Supplementary file 2 — Additional file 2. [file 40795_2022_501_MOESM2_ESM.docx]

**Arbaminch University Sawla Campus**

**Department of Food Technology and Process Engineering**

**Household Dietary diversity data questionnaire 2013**

**Identification information**

Name of surveyor_____________________ Name of survey supervisor_________________________

Name of village/community_________________ Cluster Number__________________________

Interview date ____/______/________ Rescheduled interview date____/______/_________

Time of interview ___________________ House hold code______________________________

Problems with interview (comment)__________________________________________________________

Household level consider foods eaten by any member of the household, and exclude foods purchased and eaten outside of the home. For any food groups not mentioned, ask the respondent if a food item from this group was consumed.

| No | Food group | Examples | Yes=1  No=0 |
| --- | --- | --- | --- |
| 1. | **Cereals** | Corn/maize, rice, wheat, sorghum, millet or any other grains or food made from these (e.g. Bread, noodles, porridge or other grain products) and other locally available grains |  |
| 2. | **Vitamin A rich vegetables and tubers** | Pumpkin, carrots, squash, or sweet potatoes that are orange inside, red sweet pepper and others locally available vitamin A rich vegetables. |  |
| 3. | **White tubers and Roots** | White Potatoes, white yams, white cassava, or other foods made from roots |  |
| 4. | **Dark green leafy vegetables** | Dark green/leafy vegetables, including wild ones and locally available vitamin A rich leaves such as amaranth, cassava leaves, kale, spinach etc. |  |
| 5. | **Other vegetables** | Other vegetables E.g. tomato, onion, eggplant including wild vegetables |  |
| 6. | **Vitamin A rich fruits** | Ripe mangoes, ripe papaya, dried peaches and other locally available vitamin A rich fruits |  |
| 7. | **Other fruits** | Other fruits including wild fruits |  |
| 8. | **Organ meat (Iron rich)** | Liver, kidney, heart or other organ meats or blood-based foods |  |
| 9. | **Flesh meats** | Beef, pork, lamb, goat, chicken and sheep |  |
| 10. | **Eggs** | Hen egg |  |
| 11. | **Fish** | Fresh or dried fish |  |
| 12. | **Legumes, Nuts and seeds** | Beans, peas, lentils, nuts, seeds or foods made from these |  |
| 13. | **Milk and milk products** | Milk, cheese, yoghurt or other milk products |  |
| 14. | **Oils and Fats** | Oil, fats or butter added to food or used for cooking |  |
| 15. | **Sweets** | Sugar, honey, sweetened soda or sugary foods such as chocolates, candies, cookies and cakes |  |
| 16. | **Spices, condiments, beverages** | Spices (black pepper, salt), condiments (soy sauce, hot sauce), coffee, tea, alcoholic beverages like *tella, tej, kineto.* |  |
|  | Did you or anyone in your household eat anything (meal or snack) Outside of the yesterday | |  |
