# Supplementary material for: Stunting and associated factors among 6–23 month old children in drought vulnerable kebeles of Demba Gofa district, southern Ethiopia
Source: BMC Nutr. 2022 Jan 26;8:9. doi: 10.1186/s40795-022-00501-2 (PMC8790906; doi:10.1186/s40795-022-00501-2)
Supplement: Supplementary file 3 — Additional file 3. [file 40795_2022_501_MOESM3_ESM.docx]

**Arbaminch University Sawla Campus**

**Department of Food Technology and Process Engineering**

**Demographic and Socioeconomic survey questionnaire 2013**

**Short Introduction:**

My name is ____________________________________, I’m from Arbaminch University Sawla campus department of Food Technology and Process Engineering. This department conducting a research entitled “Prevalence and Determinants of Stunting among 6-23 months children in Drought vulnerable kebeles of Demba Gofa district, Southern Ethiopia”. The study tries to assess demographic, socioeconomic, material and child health care environmental health condition of the community in the study area. So, you are kindly requested to be part of this survey. I will ask you few questions for 10-15 minutes. Your honest answers to these questions will help us for better understanding on the topic, and will eventually help in designing and implementing appropriate interventions to alleviate related problems.

I greatly appreciate your participation in the study.

**Identification information**

Name of surveyor_____________________ Name of survey supervisor_________________________

Name of village/community_________________ Cluster Number__________________________

Interview date ____/______/________ Rescheduled interview date____/______/_________

Time of interview ___________________ House hold code______________________________

Problems with interview (comment)__________________________________________________________

**Visiting Table**

|  | Visit 1 | Visit 2 | Visit 3 |
| --- | --- | --- | --- |
| Date |  |  |  |
| Result* |  |  |  |

*Result code 1- Complete

2- Incomplete/partially completed

3- Respondent not available

4- Other, specify_______ _______

**Part I**: **Socio- demographic and economic characteristics of households**

| **S. No** | **Question** | **Response** | **Code** | **remark** |
| --- | --- | --- | --- | --- |
| 101. | What is your ethnicity? | 1. Gofa  2. Wolaita  3. Gamo  4.Other(specify)_________ |  |  |
| 102. | What is mother’s/care giver`s age?  (in years) | _____________ |  |  |
| 103. | What is your religion? | 1. Protestant  2. Orthodox  3. Muslim  4. Catholic  5.Other(specify)_________ |  |  |
| 104. | What is your current marital status? | 1. Single  2. Married  3. Divorced  4. Widowed  5. Other (Specify)_______ |  |  |
| 105. | Did you ever attend formal education | 1. Yes 2. No |  |  |
| 106. | Your husband did attend formal education? | 1. Yes 2. No |  |  |
| 107. | What is your occupation? | 1. House wife  2. Employee  3. Merchant  4. Other(specify)______ |  |  |
| 108. | What is the occupation of your husband? | 1. Farmer  2. Employee  3. Merchant  4.Other(specify)______ |  |  |
| 109. | What is the total number of your family size that present currently? |  |  |  |
| 110. | What is the total number of under- five children in your home? |  |  |  |
| 111. | Birth interval from the immediate elder in the house | ________in years |  |  |
| 112. | Does this household own livestock? | 1.Yes  2.No |  |  |
| 113. | How much is average monthly income of your household in ETB? |  |  |  |
| 114. | Does this household have own agricultural land? | 1.Yes  2. No |  |  |

| **Part II: Maternal characteristic** | | | | | |
| --- | --- | --- | --- | --- | --- |
| 201 | Did you visit health facility for ANC during your Pregnancy for this child? | 1. Yes 2. No |  | If No 301 |  |
| 202 | If yes how many times did you visit during time of your pregnancy of this child?( No of follow up) |  |  |  |  |
| 203 | Did you get counseling on child feeding (breast and Complimentary feeding) during ANC visit of this child? | 1.Yes  2. No |  |  |  |
| 204 | Did you get counseling on self feeding during pregnancy and lactation? | 1. Yes 2. No |  |  |  |
| 205 | If yes for Q204, did you receive extra food during pregnancy? | 1.Yes  2. No |  |  |  |
| 206 | How many times did you feed breast within a day (24hrs)? |  |  |  |  |

**Part III: Child characteristics**

| 301 | Child sex | 1. Male 2. Female |  |  |
| --- | --- | --- | --- | --- |
| 302 | Child age in months (Verify child’s date of birth by observing the child’s health card or use local calendar) | __________months |  |  |
| 303 | Where did you deliver this child? | 1. Health institution 2. At home |  |  |
| 304 | Child have diarrhea in the last two weeks? | 1. Yes 2. No |  |  |
| 305 | Child have measles in the past one year | 1. Yes 2. No |  |  |
| 306 | Height of the child in (cm) | ___________cm |  |  |

**Part-IV. Child caring practices**

| 401 | Time at which breast milk initiated after delivery | 1. Within the1^st^ hour 2. After 1 hour 3. If other (Specify) |  |  |
| --- | --- | --- | --- | --- |
| 402 | Squeeze out of 1^st^ milk | 1. Yes 2. No |  |  |
| 403 | If yes, why? | 1. Not important  2. Unknowingly |  |  |
| 404 | How long did you exclusively breast feed this child? | Months |  |  |
| 405 | Child age at which you initiated complementary feeding for this child | Months |  |  |
| 406 | Did a child have worm infection? | 1. Yes 2. No |  |  |
| 407 | Did a child receive de-worming? | 1.Yes  2. No |  |  |
| 408 | Did a child receive Vitamin A supplementation? | 1. Yes  2. No |  |  |
| 409 | Vaccination status of children (Check immunization card, scar) | 1. Completed recommended for his age 2. Not Completed 3. Not at all vaccinated 4. Being vaccinating |  |  |
| 410 | Your child feed on fruits and/or Vegetables occasionally? | 1.Yes  2. No |  |  |
| 411 | Your child feed on animal source of food occasionally?  (Especially milk, poultry and meat their derivatives) | 1. Yes 2. No |  |  |

**Part V: Environmental health condition**

| 501 | What type of water source are you used for drinking? | 1. Protected spring water  2. Unprotected spring water  3. Public stand pipe(tap)  4. Other(specify) |  |  |  |  |
| --- | --- | --- | --- | --- | --- | --- |
| 502 | Is there functional hand washing facility at (near) toilet? (Check it) | 1. Full 2. Partial 3. Never at all |  |  |  |  |
| 503 | Household dietary diversity score | Will be filled in office after 24 hrs dietary recall analysis |  |  |  |  |
